# Seasonal variation and its interaction with pre-pregnancy BMI for GDM: a large population-based study in Tianjin, China

**DOI:** 10.1038/s41598-023-49609-w

**Published:** 2023-12-21

**Authors:** Weiqin Li, Leishen Wang, Jia Guo, Wei Dong, Shuang Zhang, Wei Li, Junhong Leng

**Affiliations:** Tianjin Women and Children’s Health Center, Tianjin, 300070 China

**Keywords:** Public health, Weight management

## Abstract

To evaluate the independent association of seasonal variation with GDM incidence in Tianjin, China, and to test whether there is an additive interaction between seasonal variation and pre-pregnancy body mass index (BMI) on GDM incidence. A population-based observational cohort study was conducted using the healthcare records data from Tianjin, China. Logistic regression was used to obtain odds ratios (ORs) and 95% confidence intervals (CIs). Additive interaction between pre-pregnancy BMI groups and seasons was estimated by using relative excess risk due to interaction (RERI), attributable proportion due to interaction (AP), and synergy index (S). Among the 112,639 pregnant women, 20.8% developed GDM at 24–28 weeks of gestation. The multivariable adjusted ORs and 95% CIs were 1.00, 1.00 (0.96–1.05), 1.15 (1.09–1.20) and 1.22 (1.16–1.29) respectively based on seasons (spring, summer, autumn and winter). Compared with the spring/summer and pre-pregnant BMI < 24 kg/m^2^ group, co-presence of autumn/winter and pre-pregnancy BMI ≥ 24 kg/m^2^ increased the OR from 1.00 to 2.70 (95% CI 2.28–3.20), with a significant additive interaction: RERI (0.32, 95% CI 0.19–0.45), S (1.21, 95% CI 1.12–1.31) and AP (0.11, 95% CI 0.07–0.16). Autumn/winter is an independent risk factor for GDM incidence, and can significantly amplify the obesity-associated risk for GDM incidence. The underlying mechanism warrants further investigations. We suggest that seasonality is an additional factor when interpreting OGTT results for the diagnosis of GDM.

## Introduction

Gestational diabetes mellitus (GDM) is a type of diabetes first diagnosed in the second or third trimester of pregnancy, often at 24–28 weeks of gestation. The prevalence of GDM varies from 1 to 25%, depending on different regions, populations and diagnostic criteria^1^. The International Diabetes Federation (IDF) Diabetes Atlas estimated the global and regional GDM prevalence of 2021 by International Association of Diabetes in Pregnancy Study Group's Criteria (IADPSG), and the GDM prevalence was 14.0% (95% confidence interval: 13.97–14.04%)^2^. GDM usually causes many adverse consequences, such as macrosomia^3^, preterm birth^3,4^, shoulder dystocia, birth trauma, neonatal morbidities^5–7^, higher risk for developing diabetes during their lifetime^8,9^, and higher risk of childhood obesity in their offspring^10^. As GDM patients rarely have symptoms at the time of diagnosis, screening and diagnosis of GDM are crucial.

Environmental factors can have an impact on specific metabolic status in pregnant women, leading to an increase in the effect on GDM. Therefore, identifying GDM environmental risk factors is very important^11^. Recently, season variation has been studied as one of the environmental factors affecting GDM. However, studies investigating the effect of seasons on GDM revealed controversial results. Some studies showed an increased frequency of GDM in summer than in winter in certain populations^12,13^, while others did not confirm the seasonal variation of GDM^14,15^. Reasons for the difference in associations across studies are not clear; however, the study samples differed at baseline by pre-pregnancy body mass index (BMI), health status, geographical location and ethnicity. Moreover, some previous studies had a small sample size and thus lacked adequate statistical power. In addition, few studies focused on the interaction of season variation with pre-pregnancy BMI on GDM. Using a large population-based study in Tianjin, China, the aim of the present study is to evaluate the independent association of seasonal variation with GDM incidence, and to test whether there is an additive interaction between seasonal variation and pre-pregnancy BMI on GDM incidence.

## Materials and methods

### Population and study design

Tianjin is a metropolitan city in Northern China, ranking fourth in population size (15 millions) among Chinese cities. It consists of 16 county-level administrative areas, including 6 central urban districts, 1 new urban district, 4 suburban districts, and 5 rural districts. The prenatal care in Tianjin is routine, with a three-tier care system consisting of more than 300 primary hospitals, 6 district-level Women and Children’s Health Centers (also including secondary hospitals), and a city-level Women and Children’s Health Center (also including tertiary hospitals). In Tianjin, all pregnant women are registered within the first trimester at primary hospitals where they receive antenatal care until the 28th gestational week, unless they have high-risk pregnancy. Then, they are referred to a secondary or a tertiary hospital for antenatal care till delivery. Health care records of pregnant women have been collected and been available in electronic form since 2009.

This study was a retrospective cohort study using the electronic data from health care records of pregnant women in Tianjin, China. Participants in this retrospective study were selected based on the electronic data whose last menstruation period was from July 2019 to June 2021. Of them, 99.8% pregnant women underwent the GDM screening test at the 24th–28th gestational week between March 2020 and November 2021. After excluding multiple pregnancies (n = 1374) and those without self-reported pre-pregnant BMI data which was required for this analysis (n = 186), 112,639 were included in the final analyses. The Ethics Committee for Clinical Research of Tianjin Women and Children’s Health Center approved the study and analysis plan (No: lw20220719). All methods were performed in accordance with the relevant guidelines and regulations. Since this is a retrospective analysis of data routinely collected from patients, the written informed consent was not applicable. The Ethics Committee for Clinical Research of Tianjin Women and Children’s Health Center agreed to waive the need for written informed consent from all participants involved in our study.

### Measurements

The Pregnant Women Health Records include general information which is self-reported at the first antenatal care visit (birth date, ethnicity, education, pre-pregnancy body weight, abortion history, last menstrual period, smoking and drinking habits, etc.), clinical measurements (height, weight, and blood pressure for each antenatal care visit, gynecological examinations, ultrasonography, gestational diabetes screening result at 24–28 gestational week, and other lab tests), pregnancy outcomes (date of delivery, delivery modes, labor complications, etc.), and postnatal period examinations (< 42 days after delivery).

Maternal age at first antenatal care visit was calculated as the period in years from the date of birth to the date of first antenatal care visit. Family history of diabetes was defined as having one or more first degree relatives with diabetes. Education attainment was classified into 2 categories: > 12 years of schooling and ≤ 12 years of schooling. The pre-pregnant body weight was recalled at the first antenatal visit at 12.2 (median 11.7) weeks of gestation.

The weight and height at the first antenatal visit were measured in light clothing and without shoes by using a beam balance scale (RGZ-120, Jiangsu Suhong Medical Instruments Co., China). BMI was calculated by dividing weight in kilograms by the square of height in meters and was categorized as underweight (BMI < 18.5 kg/m^2^), normal weight (BMI 18.5–< 24 kg/m^2^), overweight (BMI 24–< 28 kg/m^2^), or obese (BMI ≥ 28 kg/m^2^) according to the standard of Working Group on Obesity in China^16^. Sitting blood pressure (BP) was measured after at least 10 min rest by using a calibrated mercury sphygmomanometer (XJ11D, Shanghai Medical Instruments Co., China) at the first antenatal care visit. BP was measured twice with a 5-min interval between measurements and the mean value was used.

Pregnant women living in Tianjin have participated in routine screening for GDM since 1999 (3), and the screening rate has been about 90% since 2014. Two-step method was adopted for GDM screening using the IADPSG criteria. First, all pregnant women at 24–28 weeks of gestation participated in a fasting glucose screening test, and pregnancy women received different suggestions according to the results. Those with fasting blood glucose (FPG) ≥ 5.1 mmol/L can be directly diagnosed as GDM, and 75-g 2-h oral glucose tolerance test (OGTT) is not necessary; those with FPG < 4.4 mmol/L are considered to be at low risk of developing GDM, and OGTT can be temporarily avoided; those with FPG 4.4–< 5.1 mmol/L should perform OGTT as soon as possible, and GDM is diagnosed according to IADPSG criteria: FPG ≥ 5.1 mmol/L, 1-h blood glucose ≥ 10.0 mmol/L, or 2-h blood glucose ≥ 8.5 mmol/L. Plasma glucose levels were measured using an automatic analyzer (TBA-120FR; Toshiba, Japan).

Seasons are determined based on GDM screening dates, for spring (March to May), summer (June to August), autumn (September to November) and winter (December to next February).

### Statistical analyses

The criterion of statistical significance was < 0.05 (for two-sided tests). All statistical analyses used IBM SPSS Statistics 20.0 (IBM SPSS, Chicago, IL). The general characteristics between GDM group and Non-GDM group were compared by using student’s *t*-test for continuous variables if their normal distribution was not rejected, and *Chi-square* test for categorical variables.

Binary logistic regression models were used to obtain odds ratios (ORs) and their 95% confidence intervals (CIs) of pre-pregnancy BMI and seasons as categorical variables (pre-pregnancy BMI: < 18.5, 18.5 ≤ BMI < 24 [reference], 24 ≤ BMI < 28, and ≥ 28 kg/m^2^; seasons: spring [reference], summer, autumn and winter) with the risks of GDM. We included three models in the logistic analyses: Model 1, Univariate analyses. Model 2, adjusted for age, height, education, Han-ethnicity, cigarette smoking, alcohol drinking, family history of diabetes, variables at first antenatal care visit (fasting glucose level, and systolic blood pressure), and variables at GDM screening (gestational age and weight gain). Model 3, adjusted for variables in model 2 and also seasons in pre-pregnancy BMI analysis, and pre-pregnancy BMI in seasons analysis.

The additive interaction between maternal pre-pregnancy BMI and seasons on GDM was further tested. Three indicators were used to judge whether the additive interaction was statistically significant, including relative excess risk due to interaction (RERI), attributable proportion due to interaction (AP), and synergy index (S) (using an Excel calculator for additive interaction which was available at http://www.epinet.se)^17^. Briefly, RERI is the excess risk due to interaction relative to the risk without exposure. AP refers to the attributable proportion of disease that is due to interaction among individuals with both exposures. S is the excess risk from both exposures when there is an additive interaction, relative to the risk from both exposures without interaction. Significant RERI > 0 or AP > 0 or S > 1 indicated an additive interaction, i.e., seasons enhanced the effect of obesity on GDM incidence. When using the Excel calculator, the two exposure factors should be binary categorical variables. So pre-pregnancy BMI were divided into two groups (pre-pregnancy BMI < 24 kg/m^2^ as the non-exposure group, and pre-pregnancy BMI ≥ 24 kg/m^2^ as the exposure group), and seasons were divided into two groups (spring/summer as the non-exposure group, and autumn/winter as the exposure group).

## Results

Among the 112,639 women, the mean age was 29.8 ± 4.4 years, 95.5% were Han-ethnicity, 20.8% were overweight and 9.1% were obese. Totally, 26.4%, 31.6%, 26.8% and 15.1% of pregnant women underwent GDM screening at spring, summer, autumn and winter, respectively. Among them, 20.8% (n = 23,454) developed GDM. Compared with non-GDM women, those with GDM were older, shorter and lower educated, had higher pre-pregnancy BMI, higher fasting glucose level and systolic/diastolic blood pressure at first antenatal care visit, larger gestational age and weight gain at GDM screening (all *P* < 0.05). GDM women were more likely to be cigarette smokers and alcohol drinkers, and to have family history of diabetes than those without GDM (Table 1).

**Table 1:** Characteristics of subjects according to occurrence of gestational diabetes mellitus.

	All participants	Non-GDM	GDM	*P*-value
No. of subjects	112,639	89,185	23,454	
Age, years	29.8 ± 4.4	**29.5 ± 4.4**	**30.8 ± 4.6**	**< 0.001**
Height, cm	162.6 ± 5.1	**162.6 ± 5.1**	**162.5 ± 5.1**	**0.007**
Education > 12 years	71,521 (69.1)	**57,727 (70.4)**	**13,794 (64.0)**	**< 0.001**
Han-ethnicity	107,580 (95.5)	85,135 (95.5)	22,445 (95.7)	0.116
Pre-pregnancy BMI, kg/m^2^	22.6 ± 3.8	**22.2 ± 3.6**	**24.3 ± 4.3**	**< 0.001**
Pre-pregnancy BMI group, kg/m^2^				** < 0.001**
< 18.5	11,641 (10.3)	**10,460 (11.7)**	**1181 (5.0)**	
18.5–< 24	67,349 (59.8)	**55,923 (62.7)**	**11,426 (48.7)**	
24–< 28	23,382 (20.8)	**16,647 (18.7)**	**6735 (28.7)**	
≥ 28	10,267 (9.1)	**6155 (6.9)**	**4112 (17.5)**	
Cigarette smoking	821 (0.7)	**595 (0.7)**	**226 (1.0)**	**< 0.001**
Alcohol drinking	91 (0.1)	**64 (0.1)**	**27 (0.1)**	**0.038**
Family history of diabetes	3790 (3.4)	**2500 (2.8)**	**1290 (5.5)**	** < 0.001**
**Variables at first antenatal care visit**				
Gestational age, weeks	12.2 ± 3.9	12.2 ± 3.9	12.2 ± 3.8	0.352
Fasting glucose level, mmol/L	4.8 ± 0.7	**4.7 ± 0.6**	**5.1 ± 0.8**	** < 0.001**
Systolic blood pressure, mmHg	110.3 ± 11.1	**109.5 ± 10.8**	**113.5 ± 11.6**	**< 0.001**
Diastolic blood pressure, mmHg	71.4 ± 8.2	**70.8 ± 8.0**	**73.6 ± 8.6**	**< 0.001**
**Variables at GDM screening**				
Gestational age, weeks	25.4 ± 1.7	**25.4 ± 1.7**	**25.5 ± 2.0**	**< 0.001**
Seasons at GDM screening				** < 0.001**
Spring	29,792 (26.4)	**23,668 (26.5)**	**6124 (26.1)**	
Summer	35,650 (31.6)	**28,621 (32.1)**	**7029 (30.0)**	
Autumn	30,211 (26.8)	**23,805 (26.7)**	**6406 (27.3)**	
Winter	16,986 (15.1)	**13,091 (14.7)**	**3895 (16.6)**	
Weight gain*, kg	7.3 ± 4.1	**7.3 ± 3.9**	**7.4 ± 4.5**	**0.013**

Data are reported in mean ± SD or number (%). Significant values are in bold.

*GDM* gestational diabetes.

*Weight gain was calculated by weight at GDM screening minus self-reported pre-pregnancy weight.

**Figure 1:**
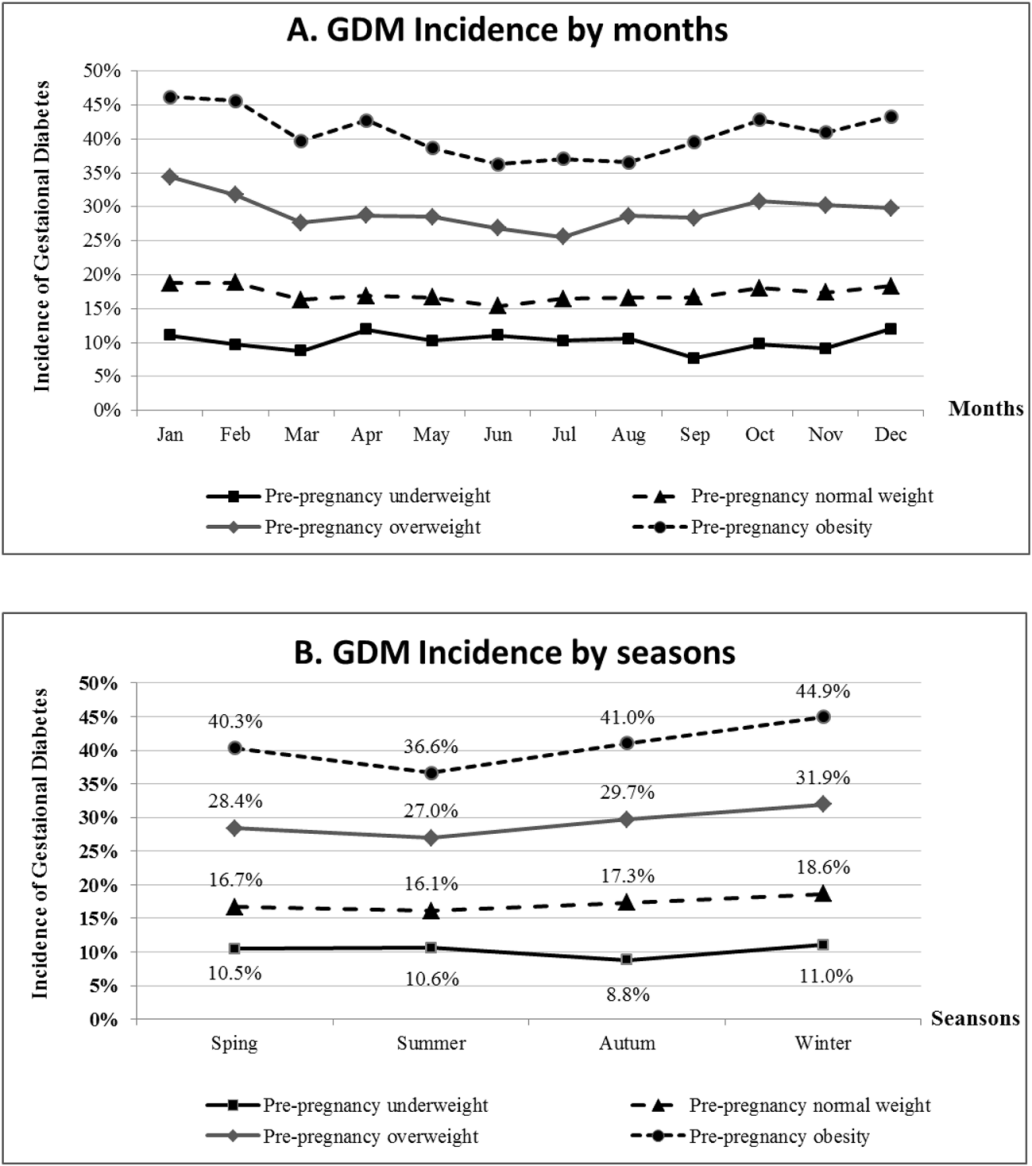
Incidence of gestational diabetes (GDM) according to months (**A**) and seasons (**B**) stratified by pre-pregnancy body mass index (BMI) groups.

GDM incidence according to months and seasons stratified by pre-pregnancy BMI groups were shown in Fig. 1A,B. GDM incidence was lowest in summer, higher in autumn, and highest in winter among women with pre-pregnant normal weight, overweight and obesity, but not among women with pre-pregnant underweight. The values of weight gain at GDM screening were lowest in summer, higher in spring and autumn, and highest in winter in all pre-pregnancy BMI groups (Appendix Figure).

There were significant association of GDM with different groups of pre-pregnancy BMI and seasons in both univariate (Model 1) and multivariate (Model 2) analyses (Table 2). When pre-pregnancy BMI and seasons entered the multivariable-adjusted model simultaneously (Model 3), both pre-pregnancy BMI and seasons were independently associated with GDM incidence. The multivariable adjusted ORs and 95 CIs were 0.67 (0.62–0.71), 1.00, 1.59 (1.53–1.66) and 2.17 (2.06–2.30) based on different levels of pre-pregnancy BMI groups (< 18.5, 18.5–< 24, 24–< 28 and ≥ 28 kg/m^2^), and 1.00, 1.00 (0.96–1.05), 1.15 (1.09–1.20) and 1.22 (1.16–1.29) based on seasons (spring, summer, autumn and winter), respectively.

**Table 2:** Odds ratios and 95% confidence intervals of gestational diabetes by different groups of pre-pregnancy BMI and seasons.

	No. of subjects	No. of cases	Crude prevalence (%)	Odds ratios (95% confidence intervals)		
				Model 1	Model 2	Model 3
Pre-pregnancy BMI group, kg/m^2^						
< 18.5	11,641	1181	10.1	**0.55 (0.52–0.59)**	**0.66 (0.62–0.71)**	**0.67 (0.62–0.71)**
18.5–< 24	67,349	11,426	17.0	1.00	1.00	1.00
24–< 28	23,382	6735	28.8	**1.98 (1.91–2.05)**	**1.60 (1.54–1.66)**	**1.59 (1.53–1.66)**
≥ 28	10,267	4112	40.1	**3.27 (3.13–3.42)**	**2.19 (2.07–2.31)**	**2.17 (2.06–2.30)**
Seasons						
Spring	29,792	6124	20.6	1.00	1.00	1.00
Summer	35,650	7029	19.7	0.95 (0.91–0.99)	1.00 (0.96–1.05)	1.00 (0.96–1.05)
Autumn	30,211	6406	21.2	**1.04 (1.00–1.08)**	**1.17 (1.12–1.23)**	**1.15 (1.09–1.20)**
Winter	16,986	3895	22.9	**1.15 (1.10–1.20)**	**1.23 (1.17–1.30)**	**1.22 (1.16–1.29)**

Significant values are in bold.

Model 1, Univariate analyses.

Model 2, Adjusted for age, height, education, Han-ethnicity, cigarette smoking, alcohol drinking, family history of diabetes, variables at first antenatal care visit (gestational age, fasting glucose level, and systolic blood pressure), and variables at GDM screening (gestational age and weight gain).

Model 3, Adjusted for variables in model 2 and also seasons (in pre-pregnancy BMI analyses), and pre-pregnancy BMI (in seasons analysis).

**Figure 2:**
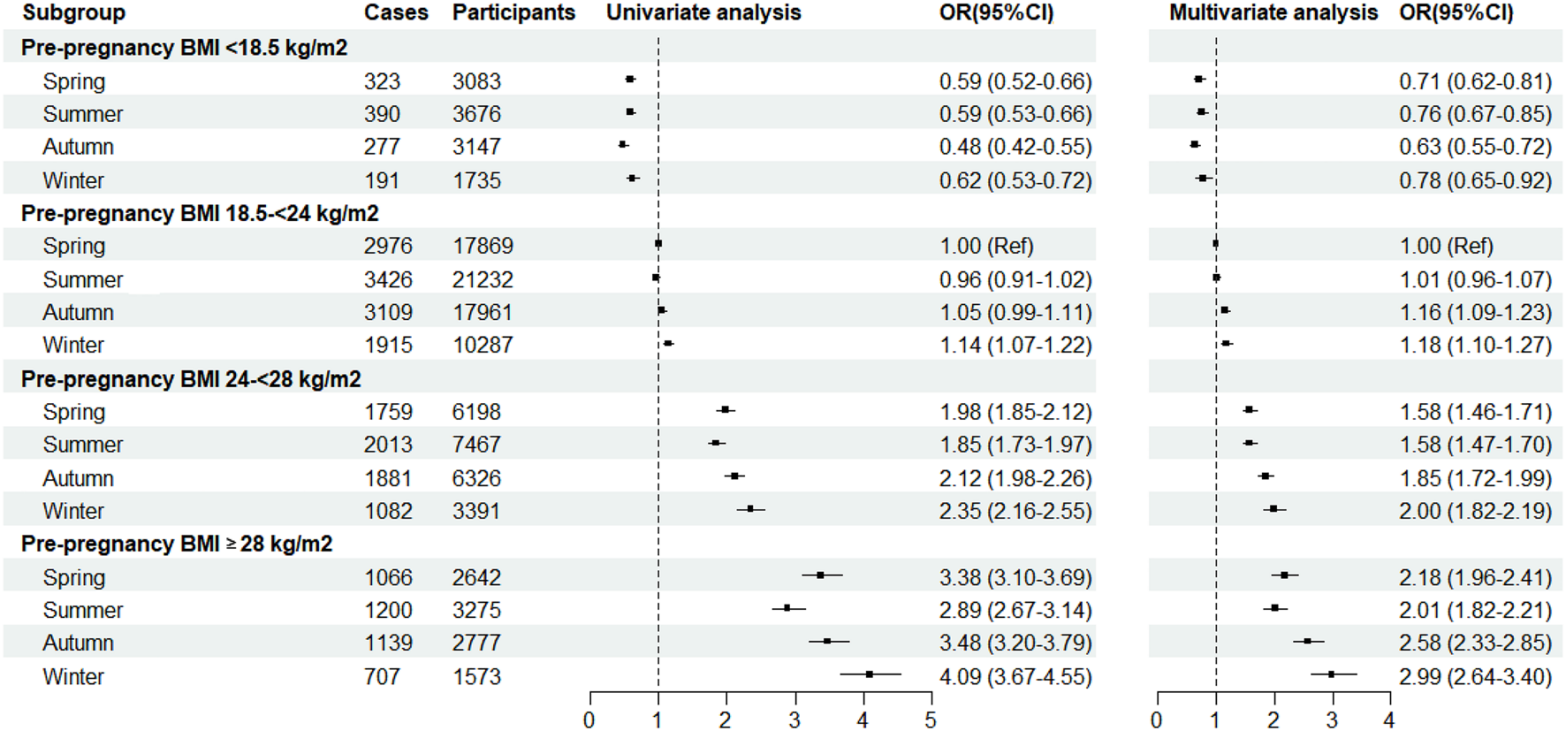
Odds ratios and 95% confidence intervals of gestational diabetes by seasons stratified by pre-pregnancy BMI groups. Multivariate analyses, adjusted for age, height, education, Han-ethnicity, cigarette smoking, alcohol drinking, family history of diabetes, variables at first antenatal care visit (fasting glucose level, and systolic blood pressure), and variables at GDM screening (gestational age and weight gain).

Figure 2 showed the association of GDM with seasons stratified by pre-pregnancy BMI groups. In general, the risk of GDM was lowest in spring or summer, higher in autumn, and highest in winter among women who were pre-pregnant normal weight, overweight and obese.

**Figure 3:**
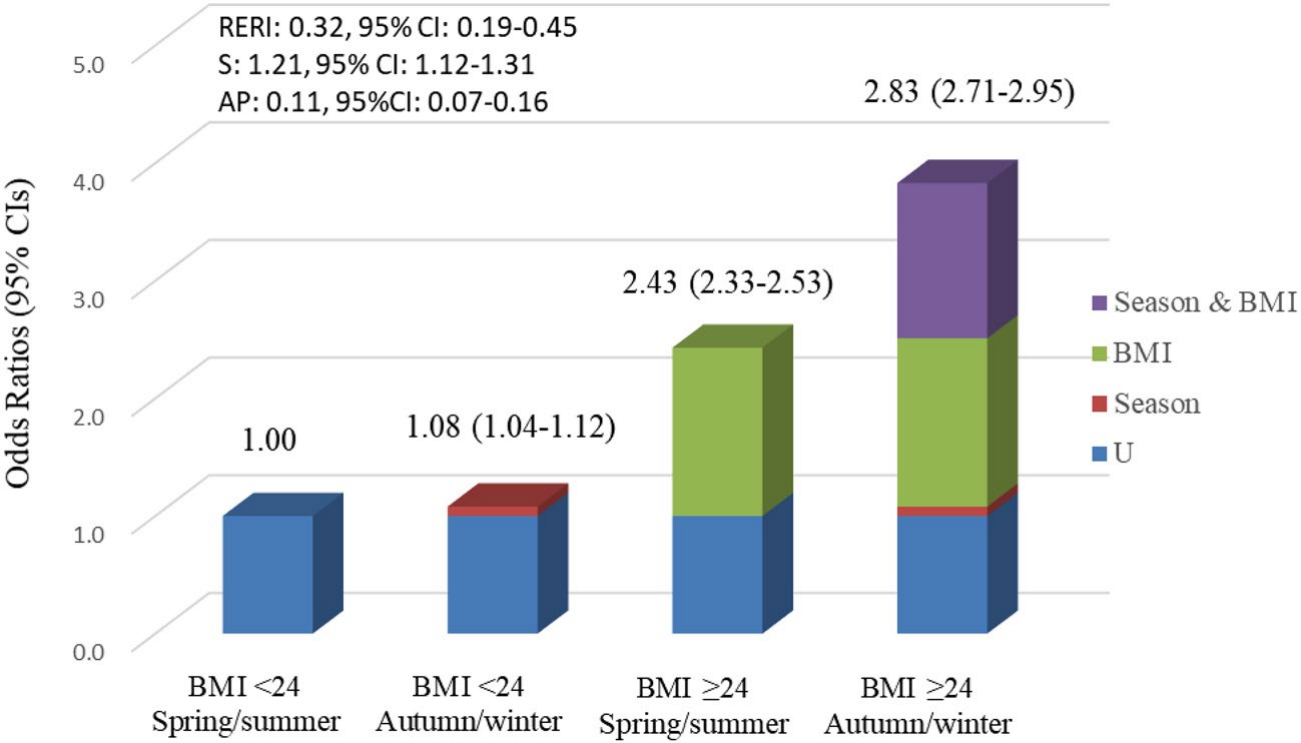
Odds ratios of gestational diabetes (GDM) with contributions from pre-pregnancy body mass index (BMI) and seasons and both. RERI represented the excess risk was 32% due to the interaction, S represented the similar meaning with RERI, while AP represented 11% of all GDM cases attributable to the interaction of pre-pregnancy BMI ≥ 24 kg/m^2^ and autumn/winter.

Figure 3 showed the additive interaction between maternal pre-pregnancy BMI and seasons on GDM. Using the spring/summer and pre-pregnant BMI < 24 kg/m^2^ group as the referent group, autumn/winter increased the OR of GDM from 1.00 to 1.08 (1.04–1.12) for pre-pregnancy BMI < 24 kg/m^2^ women, and from 2.43 (95% CI 2.33–2.53) to 2.83 (95% CI 2.71–2.95) for pre-pregnancy BMI ≥ 24 kg/m^2^ women. There is a positive additive interaction between seasonal variation and pre-pregnancy BMI on GDM incidence. RERI represented the excess risk was 32% (0.32, 95% CI 0.19–0.45) due to the interaction, S (1.21, 95% CI 1.12–1.31) represented the similar meaning with RERI, while AP represented 11% (0.11, 95% CI 0.07–0.16) of all GDM cases attributable to the interaction of pre-pregnancy BMI ≥ 24 kg/m^2^ and autumn/winter.

## Discussion

In this large population-based study in Tianjin, China, we found that autumn/winter was an independent risk factor for GDM incidence, with significantly amplifying the obesity-associated risk for GDM incidence.

Several studies have evaluated the association between seasons and GDM incidence, and results are controversial. Since a prospective study from Brazil showed an increased frequency of GDM in summer than in winter in 1994 for the first time^18^, similar results have been reported by some studies in southern Europe^12,19^, Australia^13^ and Canada^20^. However, some other studies in the UK did not confirm the seasonal variation of GDM^14,15^. A recent meta-analysis reviewed 11 studies related to the association between ambient temperature/season changes and GDM, suggested that season changes showed a significant positive relationship with GDM; however, subgroup analyses based on seasons showed the non-significant effects of spring, summer and autumn to winter on risk of GDM (p < 0.001)^21^. There was significant heterogeneity across studies, and the study samples differed on ethnicity, pre-pregnancy BMI, health status, and geographical location. To our knowledge, there was no study conducted in East Asia focusing on the seasonal variation of GDM incidence. Thus, the association between seasons and GDM is still unclear. Our study, based on a large population-based study in Tianjin, China, suggested a different result that GDM incidence was lowest in summer, higher in autumn, and highest in winter among women with pre-pregnant normal weight, overweight and obesity, but not among women with pre-pregnant underweight.

The seasonal variation of glycosylated hemoglobin (HbA1c) also attracted attention over the past ten years. Studies in the United States^22^, South Korea^23^, and Japan^24^ have observed seasonal changes that HbA1c values were higher in winter and lower in summer. The diagnosis of type 2 diabetes is also more likely in the winter months^25^. Our study showed similar seasonal variation for GDM incidence. Totally, the risk of GDM increased 15% in autumn and 22% in winter as compared with in spring. This seasonal pattern follows trends similar to those of many physiologic markers, cardiovascular and diabetes outcomes and mortality.

Studies showed that excessive gestational weight gain before GDM screening is a risk factor for GDM^26,27^, and the risk of GDM increases with the increase in gestational weight gain^28^, which may be due to the gestational weight gain more of an increase in fat than lean mass^29^. Our results revealed that seasonal changes in gestational weight gain were consistent with seasonal trends in GDM incidence in all pre-pregnancy BMI groups. This suggested that the seasonal variation in GDM incidence is largely due to the seasonal variation in gestational weight gain. However, when gestational weight gain was further adjusted, seasonal variation was an independent risk factor for GDM incidence.

The mechanism on the association between seasons and GDM incidence is still unknown. Physiological or metabolic factors related to temperature may be the main causes of seasonal fluctuations in blood glucose levels. It is known that certain hormones have seasonal variations, such as glucagon, cortisol, epinephrine, thyroid hormones and so on; these variations might affect insulin secretion or insulin resistance^30^. Other factors may need to be considered in determining the variation in GDM incidence, including the duration of daylight hours and the possible effect of these hours on light sensitive hormones (vitamin D, melatonin, and serotonin)^31^. Aside from seasonal biological variations, lifestyle or cultural events may influence the seasonal variability in glucose. People tend to have fewer outdoor activities and less exercise in winter due to cold temperature and less sunshine. They also tend to increase calorie intake in winter and lose weight in summer. Furthermore, the traditional Chinese festival "Spring Festival" is in winter, so people are more likely to eat high-calorie food during the period. These non-biological factors should be considered when evaluating seasonal variations in GDM incidence.

Our study found a significant additive interaction between pre-pregnancy BMI and seasons on GDM incidence. Autumn/winter increased the OR of GDM from 2.43 to 2.83 for pre-pregnancy BMI ≥ 24 kg/m^2^ women with the excess risk 32% due to the interaction; the other indicator AP represented about 11% of all GDM cases were attributable to this additive interaction. The mechanism on the synergistic effect of pre-pregnancy BMI ≥ 24 kg/m^2^ and autumn/winter on GDM incidence is unknown. Several lifestyle and psychosocial factors associated with seasonality, pre-pregnancy BMI may be influential in the pathophysiologic mechanisms of GDM, such as ambient temperature, physical activity, nutrient intake, and vitamin D levels^13^. Further investigations are needed to explain the reason.

More women were diagnosed of GDM in autumn/winter than in spring/summer. This study could not explain whether women were over-diagnosed in autumn/winter or underdiagnosed in spring/summer. But the results suggest that seasonality is an additional factor when interpreting OGTT results for the diagnosis of GDM, and provide a new direction for future research into the seasonal adjustment of OGTT results.

The strength of this study is that to our knowledge, it is the first study conducted in East Asia using an ethnically representative population, which focused on the association of seasonal variation with GDM incidence. It is also the first study to evaluate the interaction of seasonal variation with pre-pregnancy BMI on GDM incidence. Furthermore, this study is based on a government-managed healthcare system covering all community populations in Tianjin, China. Multi-level monitoring of data collection and quality control makes the data representative and objective. Our study had limitations. First, the IADPSG recommends using a one-step OGTT approach to identify GDM cases, whilst our antenatal care system had been using a two-step procedure to detect GDM since 1999. Thus, some GDM cases might not be diagnosed due to exclusion during this two-step procedure. Second, pre-pregnancy weight was self-reported at the first antenatal care visit, which might produce recall bias and lead to underestimations. Third, even though our analyses were adjusted for an extensive set of confounding factors, residual confounding due to unmeasured factors cannot be excluded, including chronic diseases and/or medication, employment, marital status, living alone or co-habiting, etc. Finally, participants in this study underwent the GDM screening test between March 2020 and November 2021, and so the number of participants in winter was smaller than in other seasons. To balance the distribution of seasons, we did some sensitivity analyses by only including participants who underwent the GDM screening test between August 2020 and July 2021 (a whole year, 18,626 women in spring, 17,158 in summer, 17,231 in autumn and 16,783 in winter), and results did not change.

In conclusion, as the first study conducted in East Asia, our study found that autumn/winter was an independent risk factor for GDM incidence, and could significantly amplify the obesity-associated risk for GDM incidence in northern China. The underlying mechanism warrants further investigations. We suggest that seasonality is an additional factor when interpreting OGTT results for the diagnosis of GDM in northern China.

### Supplementary Information

Supplementary Information.

## Data Availability

The datasets used and/or analyzed during the current study are available from the corresponding author on reasonable request.
