# Supplementary material for: Seasonal variation and its interaction with pre-pregnancy BMI for GDM: a large population-based study in Tianjin, China
Source: Sci Rep. 2023 Dec 21;13:22837. doi: 10.1038/s41598-023-49609-w (PMC10739738; doi:10.1038/s41598-023-49609-w)
Supplement: Supplementary file 1 — Supplementary Information. [file 41598_2023_49609_MOESM1_ESM.docx]

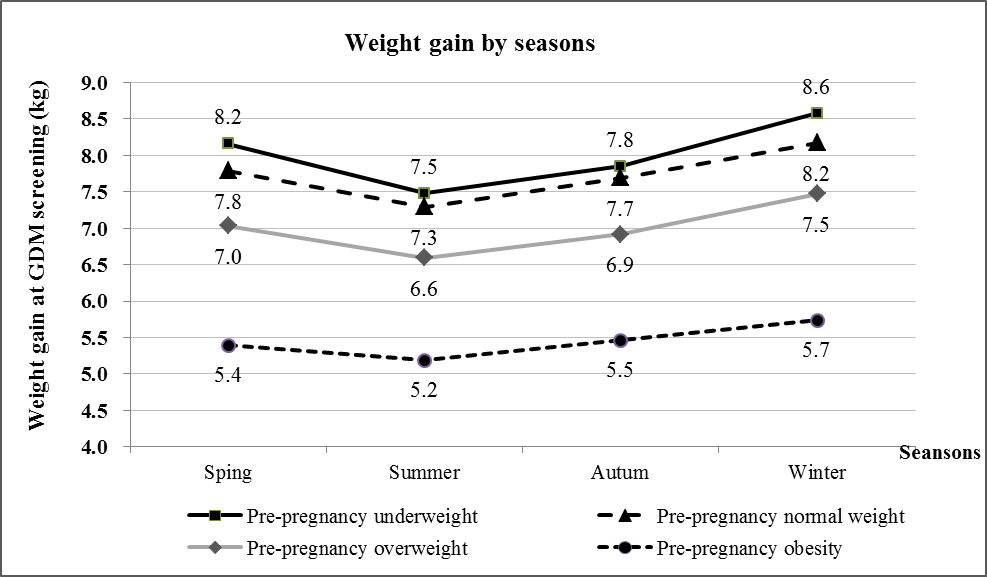


Appendix figure. Weight gain at gestational diabetes (GDM) screening according to seasons stratified by pre-pregnancy body mass index groups (BMI).
